# APP-BP1 inhibits Aβ42 levels by interacting with Presenilin-1

**DOI:** 10.1186/1750-1326-2-3

**Published:** 2007-02-07

**Authors:** Yuzhi Chen, Angela M Bodles, Donna L McPhie, Rachael L Neve, Robert E Mrak, W Sue T Griffin

**Affiliations:** 1 Department of Geriatrics, University of Arkansas for Medical Sciences, Little Rock, AR 72205, USA; 2 Department of Neurobiology & Developmental Sciences, University of Arkansas for Medical Sciences, Little Rock, AR 72205, USA; 3 Department of Psychiatry, McLean Hospital and Harvard Medical School, Belmont, MA 02478, USA; 4 Department of Pathology, University of Arkansas for Medical Sciences, Little Rock, AR 72205, USA

## Abstract

**Background:**

The β-amyloid precursor protein (APP) is sequentially cleaved by the β- and then γ-secretase to generate the amyloid β-peptides Aβ40 and Aβ42. Increased Aβ42/Aβ40 ratios trigger amyloid plaque formations in Alzheimer's disease (AD). APP binds to APP-BP1, but the biological consequence is not well understood.

**Results:**

We report that when the endogenous APP-BP1 was suppressed by small interfering RNAs (siRNAs), cell-associated Aβ42 was dramatically increased in APP_695 _expressing primary neurons. The accumulation of Aβ42 was accompanied by significant increases in APP and APP-CTF in APP-BP1 siRNA expressing neurons. In contrast, APP-BP1 overexpression in primary neurons significantly decreased the levels of Aβ and endogenous APP but not APLPs. We also investigated the potential mechanism of APP-BP1-mediated APP processing. APP-BP1 co-precipitated with Presenilin-1 (PS1) in native rat brain extracts, co-migrated with the γ-secretase components in brain membrane extracts in glycerol gradient centrifugation, and colocalized in primary neurons. Further, the endogenous PS1-CTF was significantly downregulated by APP-BP1 expression.

**Conclusion:**

Our data suggest that APP-BP1 may inhibit Aβ42 production by interacting with PS1 under physiological conditions.

## Background

The amyloid precursor protein (*APP*) *b*inding *p*rotein-*1*(APP-BP1) is the regulatory subunit of the activating enzyme for the small ubiquitin-like protein Nedd8 [1,2]. APP-BP1 is homologous to the amino-terminus of E1, and binds to Uba3 which is homologous to the carboxyl terminus of E1 and contains the catalytic cysteine residue. APP-BP1 forms a heterodimer with Uba3, and together activates the small ubiquitin-like protein Nedd8 [3]. When Nedd8 is activated, it covalently modifies (neddylates) Cullin family members [4]. Cullins are the subunit of a class of ubiquitin E3 ligase complex. Neddylated Cullins may become transiently stable, resulting in enhanced ubiquitin ligase activity and increased degradation of target proteins [5].

At the cellular level, neddylation regulates cell cycle progression at two checkpoints – APP-BP1 inhibits entry into the DNA synthesis (S) phase and promotes entry into mitosis in dividing cells [2,6], presumably by neddylation of different Cullin proteins. Loss of APP-BP1 function in the Chinese hamster liver cell line, ts41 cells, results in the accumulation of cells in the S and G1 phases of the cell cycle [6]. Expressing human APP-BP1 can rescue the cell cycle defect in ts41 cells at the non-permissive temperature [2]. Knocking out the activation subunit of the Nedd8 activating enzyme, Uba3, leads to embryonic lethality at the peri-implantation stage [7]. Therefore, the APP-BP1-mediated neddylation is critical for somatic cell survival.

The function of APP-BP1 in the brain is not well understood. In primary neurons, overexpression of APP-BP1 triggers DNA synthesis followed by apoptosis [2,8]. In Alzheimer's disease (AD), the modified APP-BP1 (about 65 kDa) is elevated in the Triton-insoluble and SDS-soluble fractions [8]. Neddylation appears to be activated in AD hippocampal neurons [8]. Based on these data, we hypothesized before that APP-BP1 was detrimental to neuronal survival. However, APP-BP1 mRNAs are highly expressed in brain hippocampal pyramidal and granule cells [1]. Nedd8 and APP-BP1 proteins are also expressed in hippocampal neurons [8]. The hippocampal brain structure is critical for learning and memory and is severely damaged in AD. These observations suggest that APP-BP1 may have a regulatory function in neurons in physiological conditions.

APP-BP1 binds to the cytoplasmic domain of APP. Data from primary neuronal cultures and drosophila genetics suggest that APP signaling is at least partially transmitted through APP-BP1 [8,9]. APP undergoes cleavage within the membrane spanning region by the γ-secretase to generate Aβ. Presenilin is an integral component of the γ-secretase complex [10]. Presenilin-deficiency abolishes most Aβ genesis if not all of it [11-13]. In addition to the interactions with APP, both APP-BP1 and PS1 regulate β-catenin levels. For example, knocking out the activation subunit of the Nedd8 activating enzyme, Uba3 [7], or PS1 [14] in mice both result in β-catenin accumulation. Based on these intricate connections, here we examined if APP-BP1 played a role in APP processing. We reported that suppression of APP-BP1 by small interfering RNAs (siRNAs) in primary neurons induced a dramatic increase in Aβ42 production accompanied by increased levels of APP and APP-CTFs. We examined the possible mechanisms of APP-BP1-mediated Aβ42 inhibition and discovered that APP-BP1 expression facilitated PS1-CTF degradation.

## Results

### APP-BP1 co-precipitated with PS1 in rat brain extracts

**Figure 1:**
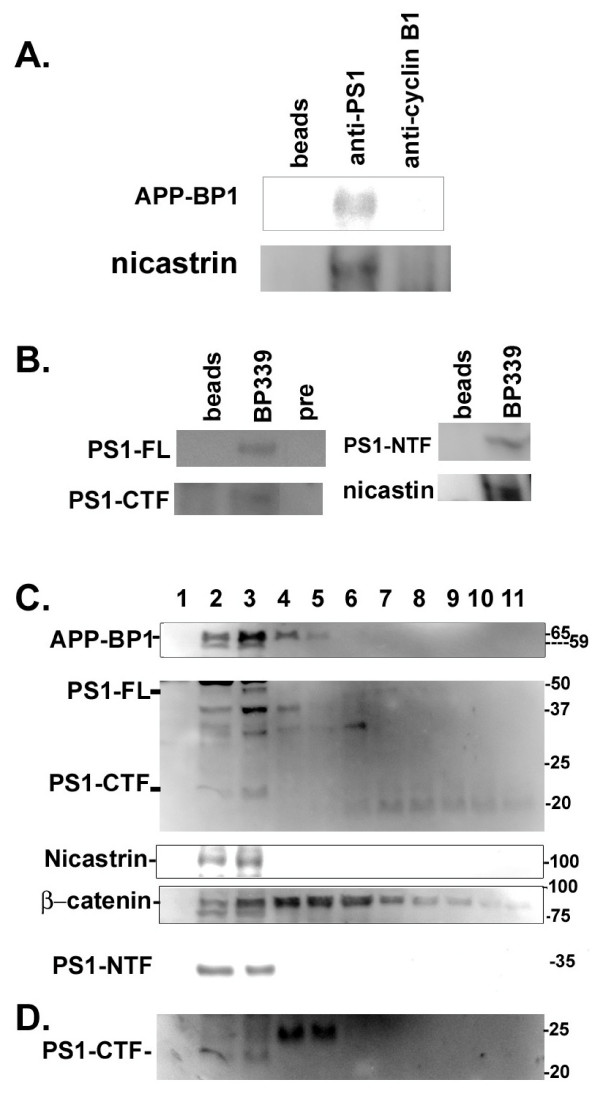
**APP-BP1, PS1 and nicastrin co-immunoprecipitated and co-migrated in brain protein extracts**. **A**. APP-BP1 co-immunoprecipitated with PS1 in brain lysates. Adult rat brain lysates were immunoprecipitated with the rabbit anti-PS1 antibody (middle lane), or a non-related antibody (rabbit anti-cyclin B1). The blot was probed with the anti-APP-BP1 antibody, BP339 or with rabbit anti-nicastrin. Anti-PS1 specifically precipitated APP-BP1 and nicastrin which were absent in control lanes. This experiment has been repeated three times. **B**. PS1 co-precipitated with APP-BP1 in membrane fractions. Proteins extracted from membrane fractions were precipitated with the APP-BP1 antibody, BP339 or with the BP339 preimmune serum (pre) or beads alone. The blot was probed with the PS1 antibody (AB5308) for PS1-FL and PS1-CTF, or with antibodies against PS1-NTF or nicastrin. **C**. APP-BP1 co-migrated with PS1 in glycerol gradient of membrane protein extracts isolated from adult rat brains. Brain membrane proteins were subjected to glycerol gradient centrifugation. Proteins in equal volumes from each fraction were resolved by SDS-PAGE gels. The blots were incubated with specific antibodies followed by ChemiGlow detections. Fractions 2 to 4 contain the majority of each protein analyzed. APP-BP1 was found to co-migrate with PS1 (FL, NTF, and CTF), and nicastrin. The location of β-catenin was also shown in the glycerol gradient. BioRad Precision Plus protein standards are shown on the right. PS1-CTF identified by AB5308 antibody was verified by a PS1-C-specific antibody (D).

PS1 and APP-BP1 both mediate the degradation of β-catenin and interact with the Aβ precursor protein APP. These connections indicated that APP-BP1 might also interact with PS1. In these experiments, rat brain PS1 was precipitated with the rabbit PS1 antibody which was raised against the PS1 loop region (AB5308, Chemicon). The immunoprecipitates were transferred to the nitrocellulose membrane and blotted with the rabbit anti-APP-BP1 antibody, BP339. The anti-PS1 antibody specifically pulled down APP-BP1 (Fig. 1A, top). In the controls where no antibody or an antibody against cyclin B1 was added to the lysate, no APP-BP1 was specifically co-precipitated. Probing the same blots with anti-nicastrin revealed that nicastrin was also in the same complex (Fig. 1A, bottom).

In reciprocal co-ip experiments, APP-BP1 was precipitated with BP339 from membrane fractions extracted from rat brains (Fig. 1B). PS1-full length (FL) and PS1-CTF probed by the anti-PS1 (AB5308) co-precipitated with APP-BP1. PS1-NTF was blotted by the PS1-NTF antibody (gift from Huaxi Xu). Fig. 1B shows that PS1 co-precipitated with APP-BP1. Similarly, nicastrin was present in the APP-BP1 immunoprecipitate as identified by anti-nicastrin blot. Based on the whole cell extracts loaded onto the same gels, we estimated that about 1% of PS1 was associated with APP-BP1. Interestingly, also about 1% of APP-BP1 was precipitated with PS1. Together, these co-ip experiments suggest that APP-BP1 is associated with the γ-secretase complex, and mutagenesis may be needed to determine the molecular interactions between APP-BP1 and the components of the γ-secretase complex.

### APP-BP1 co-migrated with PS1 in glycerol gradient

APP-BP1 mainly exists as a cytoplasmic protein about 59 kDa in crude cell fractionation analyses (Chen, unpublished observation). A small percentage of APP-BP1 that migrates at about 65 kDa can be isolated by sucrose gradient centrifugation from the Triton-insoluble and SDS-soluble brain protein extracts which are also enriched with lipids [8]. APP-BP1 post-translational modifications may allow it to interact with membrane proteins. Lipid rafts may be the site of Aβ42 genesis [15]. Lipid rafts also contain the fully assembled γ-secretase complex [16].

The PS1-containing γ-secretase complex can also be isolated from membrane fractions on a glycerol gradient [17], suggesting that the fractions from the sucrose and glycerol gradient centrifugations have overlapping resolutions. Here, we used glycerol gradient to determine if APP-BP1 co-migrated with PS1 in brain membrane protein extracts (Fig. 1C). Fraction 1 represented the top of the gradient. APP-BP1 co-migrated with PS1 after glycerol gradient centrifugation in fractions 2 to 4, which also consisted of another γ-secretase component, nicastrin. Note that the 65 kDa APP-BP1 specifically migrated with PS1 in the glycerol gradient, similar to what we previously observed in the lipid-enriched Triton-insoluble and SDS-soluble brain extracts after sucrose gradient centrifugation [8]. The fainter band right below the major band of APP-BP1 in fractions 2 and 3 was the non-modified 59 kDa APP-BP1. This 59 kDa APP-BP1 was probably reduced from the 65 kDa form due to frequent handling judging from our experience of working with these samples. The distribution of β-catenin was more spread out in the glycerol gradient with fractions 4 to 6 containing the highest amount (Fig. 1C). The distribution of PS1 was similar to those reported in glycerol gradients under similar conditions [18]. A PS1-CTF-specific antibody, PS1-C [19], also recognized the same band about 22 kDa in Fig. 1C (Fig. 1D). Together, our data suggested that the modified APP-BP1 present in a lipid-enriched membrane fraction might modulate PS1 γ-secretase activity. We are currently analyzing the type of modification that APP-BP1 may undergo for membrane targeting.

### APP-BP1 and PS1 colocalized with each other

**Figure 2:**
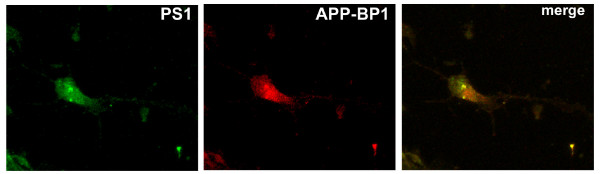
**APP-BP1 and PS1 molecules were colocalized in primary neurons**. Primary neurons were  fixed, permeablized, blocked and stained with 9E10 for mycAPP-BP1 and the  rabbit anti-PS1 (AB5308).  Primary antibodies were coupled to Alexa Fluor 594  and Alexa Fluor 488 goat secondary antibodies. The images presented were obtained under a 40× objective with a Nikon confocal microscope using the EZ-C12.20 scanning program. Independent experiments using DAPI staining along with the above two antibodies did not show nuclear co-localization of APP-BP1 and PS1 (not shown).

The PS1 and APP-BP1 antibodies used in the above experiments are well documented. To examine the interaction of APP-BP1 and PS1 even further, primary neurons were labelled with mouse anti-myc antibody for myc-APP-BP1 and rabbit anti-PS1-C. As shown in Figure 2, APP-BP1 and PS1 were colocalized as indicated by the orange color when the two colors were merged. Similar results were also obtained using the mouse anti-APP-BP1 and the rabbit anti-PS1 (AB5308) antibodies for the endogenous APP-BP1 and PS1 (not shown). APP-BP1 appeared to co-localize with PS1 outside the nucleus. Future analyses may determine the precise location of these interactions.

### Suppression of APP-BP1 by siRNAs lead to the accumulation of APP and APP-CTFs, and a dramatic increase in the levels of cell-associated Aβ42

**Figure 3:**
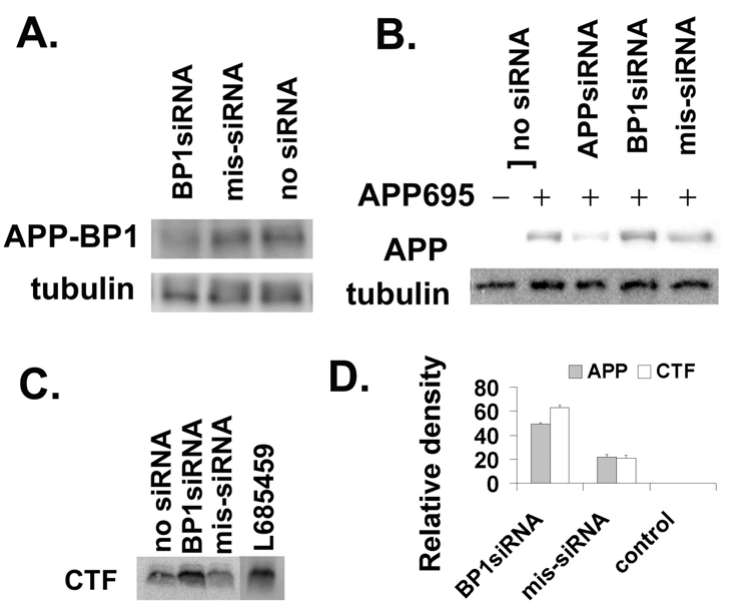
**Downregulation of APP-BP1 by siRNAs in primary neurons affected APP processing**. **A**. APP-BP1 was suppressed by APP-BP1 siRNAs but not by missense siRNAs. Primary neurons were infected with 0.5 IU of siRNA virus per cell. Equal amount of proteins extracted by RIPA buffer was analyzed by western blotting for APP-BP1 expression. The same blot was reprobed with a mouse anti-γ-tubulin antibody. No obvious changes in γ-tubulin levels were detected. *BP1siRNA, APP-BP1siRNA; mis-siRNA, misssense siRNA*. **B**. Suppression of APP-BP1 by siRNAs caused increases in exogenous human APP. Primary neurons were infected with or without APP and siRNA constructs. APP was probed with the antibody 369. The same membrane was reprobed for γ-tubulin with a mouse anti-γ-tubulin for loading controls. In each sample, 10 μg of proteins was analyzed. The data is representative of three-independent experiments. **C**. APP-BP1 siRNA inhibited APP-CTF processing in primary neurons expressing human APP. APP-CTFs were resolved on 16% Tris-Tricine gels. The blot was probed with 369. A representative of 4 independent experiments was shown. The intervening lanes between mis-siRNA and L685459 were cut off. **D**. APP-BP1 siRNA expression significantly blocked APP processing. Quantitative western blot analyses of 3 (APP) or 4 (CTF) independent experiments were carried out using two-tail *t*-Test (APP: BP1siRNA vs mis-siRNA, p < 0.003; CTF: BP1siRNA vs mis-siRNA, p < 0.001).

We showed that APP-BP1 interacted with PS1 (Fig. 1 &2), indicating that APP-BP1 might functionally regulate γ-secretase activity. To determine if APP-BP1 affected the γ-secretase cleavage of APP, we examined the effect of APP-BP1 siRNAs in APP processing by western blots and Aβ ELISA in primary neurons. Neurons were infected with human APP_695 _virus together with the APP-BP1 siRNA virus. As shown in Figure 3A, the endogenous APP-BP1 in primary neurons was suppressed by APP-BP1 siRNAs but not by the missense siRNAs. γ-Tubulin levels did not change under these experimental infection conditions.

In another set of experiments, the human APP_695 _was expressed in primary neurons with or without a siRNA virus (Fig. 3B). Immunoblots with the anti-APP antibody, 369, showed that APP siRNAs suppressed APP protein expressions. In contrast, in the presence of APP-BP1 siRNAs, APP was increased, opposite to the effect of APP siRNAs. The control missense siRNAs did not have major effects on APP metabolism. Quantitative western blot analyses revealed a significant increase in APP levels in APP-BP1 siRNA-expressing neurons (Fig. 3D, n = 3, p < 0.003, two-tail *t*-Test).

In neurons infected with APP695 with or without the APP-BP1 siRNA virus, APP-CTF levels were analyzed in 16% Tris-Tricine gels using the antibody 369. A representative blot is shown in Fig. 3C. Based on a two-tail *t*-Test, APP-CTFs significantly accumulated in the APP-BP1 siRNA-expressing cells (n = 4, p < 0.001) (Fig. 3D). The accumulation of APP-CTFs in these experimental conditions was similar to the effect of the γ-secretase inhibitor L685459 (Sigma) (Fig. 3C). Increases in APP-CTFs have been shown in familial APP mutants [20] and in PS1 experimental deletion mutants [21]. Accumulation of APP and APP-CTFs in our experiments indicated that APP-BP1 might modulate APP cleavage by the γ-secretase.

**Figure 4:**
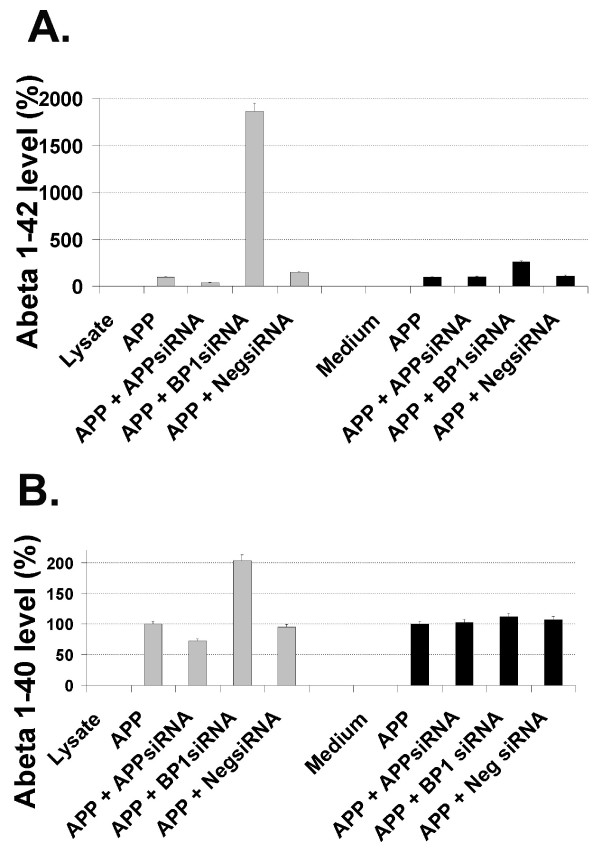
**Suppression of APP-BP1 protein expression by APP-BP1 siRNAs in primary neurons resulted in increases of intracellular Aβ42**. Intracellular (from 50 μg of protein from lysates) and secreted (from 1/15 volume of conditioned medium) Aβ42 in primary neurons were determined by Aβ42 ELISA (A). Intracellular (from 50 μg of protein) and secreted (from 1/30 volume of medium of conditioned medium) Aβ40 in primary neurons was determined by Aβ40 ELISA (B). The amount of Aβ in samples that expressed APP without any siRNA interference was used as 100% of Aβ production. All the rest of the samples were normalized to this sample, and presented as percentage in respective experimental conditions. Data is representative of three independent experiments. *BP1siRNA, APP-BP1 siRNA; NegsiRNA, Negative siRNA (Stratagene)*.

To determine if APP-BP1 affected the γ-secretase cleavage of APP, neurons were infected with APP_695 _with or without a siRNA virus. The levels of cell-associated and secreted Aβ40 and Aβ42 was analyzed by ELISA. Aβ levels were normalized to the sample that expressed only APP_695 _(100%) (Fig. 4A&B). In the presence of APP-BP1 siRNAs, the cell-associated Aβ42 was dramatically increased, but Aβ40 was elevated to a much lesser extent (Fig. 4A&B, left, respectively). Increases in cell-associated Aβ42 were accompanied by higher Aβ42 secretions in the medium (Fig. 4A, right). Aβ40 secretion was largely unaffected (Fig. 4B, right). These results obtained with the ELISA kits from Biosource International were confirmed in independent experiments using the Aβ ELSIA kits from the Genetics Company. These data suggested that APP-BP1 might specifically inhibit APP cleavage into Aβ42.

### APP-BP1 expression resulted in a reduction of rat endogenous APP but did not affect APLPs in primary neurons

**Figure 5:**
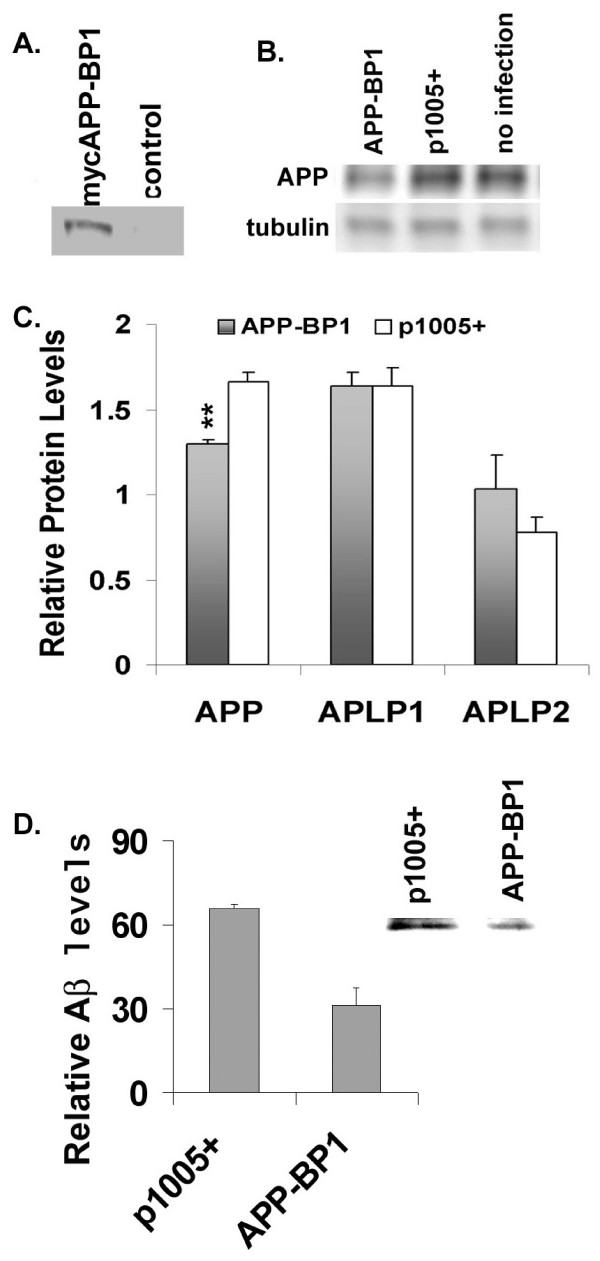
**APP-BP1 expression increased APP processing in primary neurons**. **A**. Expression of myc-tagged APP-BP1. Equal amount of total proteins from neurons infected with APP-BP1 virus or p1005+ virus was analyzed by western blotting using the mouse ant-myc antibody, 9E10. **B**. APP-BP1 overexpression downregulated rat endogenous APP. APP expression from 15 μg of proteins was analyzed by immunoblots using the anti-APP antibody 369. The amount of γ-tubulin from the same blot was reprobed with a mouse anti-γ-tubulin antibody. Representative blots from the same experiments are shown. **C**. Quantitative western blot analyses revealed that APP-BP1 significantly downregulated the endogenous APP but not APLPs in neurons overexpressing myc APP-BP1. mycAPP-BP1 expression significantly decreased APP levels compared to the sample infected with the vector (p1005+) (n = 4, p < 0.02, one-tail *t*-Test). APLP1 levels stayed the same, and APLP2 did not showed a significant change by *t*-Tests (n = 3, p = 0.1, one tail *t*-Test). **D**. RIPA buffer soluble protein extracts from primary neurons co-expressing APP-BP1 and APP_695 _or p1005+ and APP_695 _were precipitated with 6E10 followed by western blot analyses using 4G8. Mean levels of Aβ is presented in the graph (n = 4, p < 0.04). A representative of 4 independent blots was shown as an insert.

We showed that suppression of the rat endogenous APP-BP1 caused a dramatic increase in cell-associated Aβ42 (Fig. 4A, left) in addition to the accumulation of APP and CTFs (Fig. 3B–D). To further validate these APP-BP1 shRNA experiments, we inspected if overexpression of APP-BP1 in primary neurons would inhibit APP processing opposite to the effect of APP-BP1 suppression by siRNAs. In Fig. 5, a myc-tagged human APP-BP1 was expressed in primary neurons and the rat endogenous APP levels were analyzed. A representative western blot was shown in Fig. 5B. Quantitative western blot analyses showed that APP was significantly reduced in mycAPP-BP1 expressing neurons (Fig. 5C). The APP-like proteins can also undergo γ-secretase cleavage [22]. However, there were no significant changes in the levels of the APP-like proteins, APLP1 or APLP2, due to mycAPP-BP1 expression in these neurons. The decreases in the rat endogenous APP-CTFs were not analyzed because they were difficult to detect, unlike in experimental conditions where the human APP was expressed in primary neurons (Fig. 3C). The decrease in APP and not in APLPs indicated that APP-BP1 specifically affected APP cleavage although APLPs, especially APLP2, are highly homologous to APP. During our analyses, we found that APP-BP1 also interacted with APP upstream of C31 near the inner plasma membrane insertion site (see Additional file 1, Fig. 1) [1,8]. The long interacting site in APP cytoplasmic tail by APP-BP1 might be necessary for the specificity of APP-BP1.

### APP-BP1 expression inhibited cell-associated Aβ levels

We showed that APP-BP1 expression specifically downregulated rat endogenous APP (Fig. 5), but it is difficult to detect the rat endogenous Aβ levels. Here we examined if expressing human APP-BP1 can reduce the levels of cell-associated Aβ in primary neurons that co-expressed human APP_695 _Equal amount of cell extracts was precipitated by the antibody 6E10 and the precipitates were analyzed by western blotting using the antibody 4G8. The locations of Aβ peptides were determined by the standard on the same blot. Semi-quantitative analyses of Aβ levels from four co-ip experiments showed that APP-BP1 expression significantly reduced Aβ levels compared to the control (vector virus p1005+) (Fig. 5D. p < 0.04, one tail *t*-Test). A representative blot is shown as an insert (Fig. 5D). We did not differentiate Aβ42 from Aβ40 in these immuno-western blots mainly because very little Aβ40 was detected in the blots, probably because Aβ40 was very unstable in neuron extracts in contrast to Aβ42. In ELISA assays we noticed that Aβ40 was dramatically reduced after even one freeze-and-thaw cycle.

### APP-BP1 downregulated PS1-CTF stability in primary neurons

**Figure 6:**
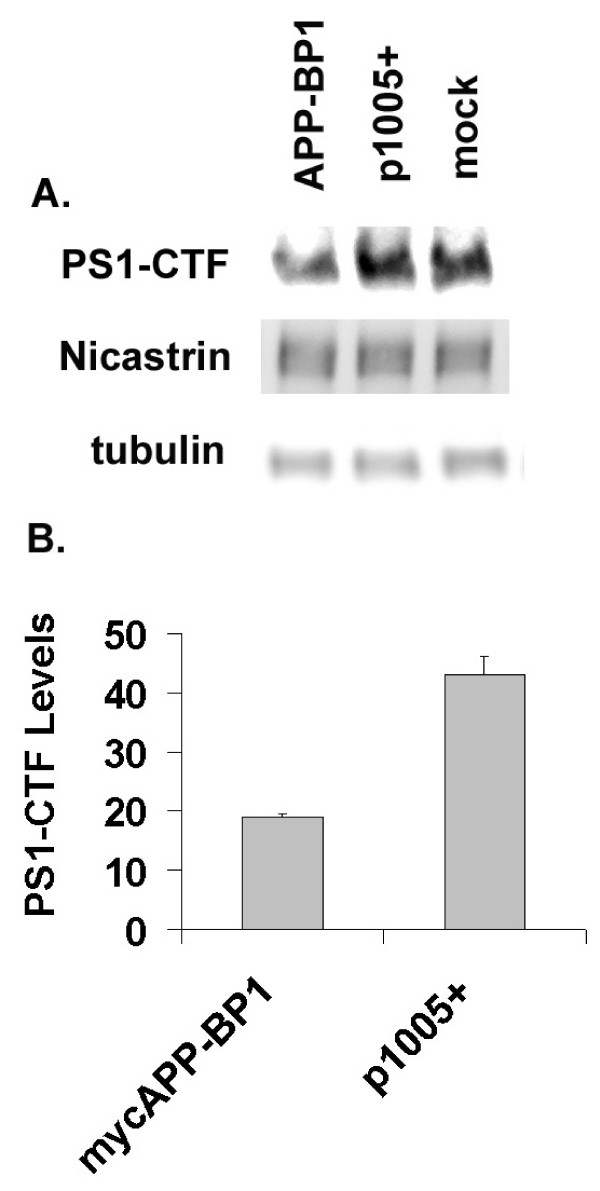
**APP-BP1 expression decreases PS1-CTF stability in primary neurons**. Primary neurons were infected with mycAPP-BP1 or p1005+ vector virus for 14 hrs before harvesting. Levels of the rat endogenous PS1 and PS1-CTF were examined by western blots. A representative PS1-CTF blot was shown in A. The same blot was reprobed with the rabbit anti-nicastrin or mouse anti-γ-tubulin antibodies after stripping. B, Quantitative western blot analyses show significant reduction of PS1-CTF in APP-BP1 expressing neurons (n = 3, p < 0.049, one tail *t*-Test).

Our data suggest that APP-BP1 functionally interacts with PS1 by inhibiting Aβ42 levels. PS1-CTF has been shown to regulate Aβ42 production through stability and endocytosis [23]. Moreover, APP-BP1-activated neddylation plays a pivotal role in protein turnover. Here the levels of PS1-CTF were examined in primary neurons overexpressing APP-BP1. Quantitative analyses revealed that PS1-CTF was significantly downregulated by APP-BP1 (Fig. 6B, n = 3, p < 0.049, one-tail *t*-Test). A representative blot was shown in Fig. 6A. In contrast, even though nicastrin co-precipitated with APP-BP1 (Fig. 1), its levels remained unchanged with APP-BP1 expression (Fig. 6A). γ-Tubulin expression was used as the loading control (Fig. 6A).

## Discussion

One of the hallmarks of AD pathogenesis is Aβ aggregation and deposition in the extra cellular space in the brain [24-26]. Increased Aβ42/Aβ40 ratios seem to initiate the disease process [27,28]. The necessity of Aβ42 for amyloid deposition is further demonstrated in mice expressing Aβ42 but not in those expressing Aβ40 [29]. However, evidence also suggests that decreases in Aβ40 may perturbs the Aβ42/Aβ40 ratio triggering the deposition of Aβ [30-32]. Familial AD APP mutations produce different levels of Aβ42, with the highest amount of Aβ42 produced by the London mutation or mutations close to the γ cleavage site in APP [33,34].

**Figure 7:**
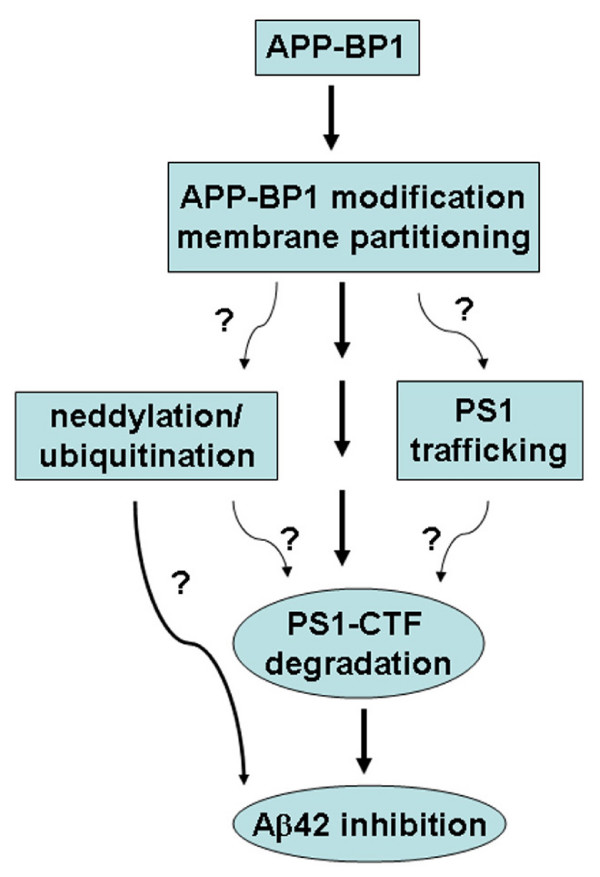
**Diagram of APP-BP1-mediated Aβ42 inhibition**. Potential pathways through which APP-BP1 inhibits Aβ42 are presented with a question mark.

In this report, we provided evidence that APP-BP1 might inhibit Aβ42 levels by interacting with PS1. We showed that APP-BP1 co-precipitated and co-migrated with the PS1 and nicastrin in brain protein extracts (Fig. 1). Native APP-BP1 and PS1 molecules were colocalized in primary neurons (Fig. 2). The significance of the APP-BP1/PS1 interaction was further investigated in primary neurons using HSV vector-mediated gene transfers. Downregulation of APP-BP1 by siRNAs in primary neurons revealed that APP-BP1 might regulate APP processing into Aβ42 (Fig. 4). Further, APP-BP1 overexpression in primary neurons resulted in the specific downregulation of rat endogenous APP, and also appeared to downregulate Aβ42 (Fig. 5). We also analyzed the potential mechanisms of APP-BP1-mediated Aβ42 inhibition and found that APP-BP1 regulated PS1-CTF levels in neurons. Together, our data suggest that APP-BP1 inhibits Aβ42 by modulating γ-secretase activity. The major findings and implications are illustrated in the diagram (Fig. 7).

All four known γ-secretase components are membrane proteins including the regulatory subunit CD147 recently reported [35-38]. The predicted molecular weight of APP-BP1 is 59 kDa. APP-BP1 is not a membrane protein, but it undergoes at least two types of posttranslational modifications that may enable membrane association [1,8]. We have shown before that an APP-BP1 (about 65 kD) specifically migrates to the lipid-enriched fraction [8]. Our new data demonstrated that the 65 kDa APP-BP1 was present in brain membrane fractions co-migrating with the γ-secretase complex, suggesting that a membrane-associated and modified APP-BP1 modulated γ-secretase activity and inhibited cell-associated Aβ42 production. APP-BP1 is extremely unstable. We could not unequivocally demonstrate that it was the 65 kDa APP-BP1 that co-precipitated with PS1 because this 65 kDa form could be easily reduced to the 59 kDa form during frequent handing in the experiments. However, glycerol gradient centrifugation (Fig. 1C) and sucrose gradient centrifugation [8] both showed that the 65 kDa APP-BP1 co-migrated with PS1. Further, our data suggest that APP-BP1 downregulates Aβ42 production by regulating PS1-CTF degradation, which indicates that APP-BP1 may affect PS1 trafficking. Determining the cellular locations of APP-BP1 and PS1 interaction may shed light on if APP-BP1 plays a role in PS1 trafficking. Further, APP-BP1 may facilitate PS1-CTF degradation by neddylation and ubiquitination. These two likely processes mediated by APP-BP1 may co-exist in a membrane compartment. A previous study showed that the small ubiquitin-like protein Sumo-2 (not Nedd8 or ubiquitin) regulates APP processing in transfected 293T cells [39]. However, this study did not specifically analyze Aβ42. APP-BP1 does have potential Sumo modification sites which may affect its function in APP processing. Detailed analyses of APP-BP1 post-translational modifications may yield clues to the partitioning of APP-BP1 to specific membrane compartments where APP-BP1 may inhibit Aβ42 genesis.

In addition to inhibiting Aβ42 genesis, our findings suggest that APP-BP1 may mediate Aβ42 degradation inside the cell. This is suggested by the observation that the accumulation of cell-associated Aβ42 greatly exceeded the amount of Aβ42 secreted into the medium in the presence of APP-BP1 siRNAs. Intraneuronal Aβ accumulation has been observed in AD [40-43], Down's syndrome [40,42,44-46], and AD mouse models [41,47-49] and is implied in AD pathogenesis (for reviews, see [25,50,51]. Due to the known function of the APP-BP1 pathway in protein degradation, APP-BP1 might also play a pivotal role in intraneuronal Aβ homeostasis.

We have documented before that APP-BP1 overexpression activates neddylation and results in neuronal death due to cell cycle reactivation [2,8]. Increases in APP-BP1 would also make it more available for post-translational modifications and the subsequent membrane partitioning with PS1 to inhibit Aβ42 genesis. Our previous data show that the predominant effect of APP-BP1 overexpression is to promote cell cycle progression in somatic cells. However, in primary neurons, APP-BP1 mainly induces neuronal death due to forced cell cycle re-entry. In these conditions, although APP-BP1 may downregulate Aβ42 production, it does not seem sufficient to overcome cell cycle-induced neuronal apoptosis due to APP-BP1 overexpression. We suspect that the Triton-soluble, cytosolic 59 kDa APP-BP1 may activate neddylation associated with cell cycle regulation which is normally inhibited by factors such as ASPP2 [52]. However, the Triton-soluble 59 kDa APP-BP1 is not increased in AD and therefore APP-BP1 may not be a direct factor inducing cell cycle events in AD [8]. This new hypothesis agrees with the observation that APP-BP1 is naturally expressed in high levels in hippocampal neurons *in vivo *[1]. Moreover, the Triton-insoluble and SDS-soluble APP-BP1, presumably the 65 kDa form, appeared to be downregulated in the brain during normal aging (See Additional file 1, Fig. 2). The combined evidence suggests that the increase of the 65 kDa APP-BP1 in AD is beneficial to neurons by inhibiting Aβ42 genesis and points to a potential therapeutic strategy of upregulating APP-BP1 modification.

Whether or not genetic variants of APP-BP1 are associated with inherited susceptibility to AD remains to be analyzed. Since APP-BP1 appears to regulate Aβ42 production, AD-susceptible APP-BP1 variants may exist. However, APP-BP1 has critical functions in somatic cell survival due to its role in neddylation. Any APP-BP1 mutations that result in neddylation dysfunction are likely to be prenatal lethal. If these two functions are independent of each other, those APP-BP1 variants which support neddylation and do not severely affect neuronal differentiation would be detectible. How neddylation and Aβ42 inhibition by APP-BP1 might be inter-related in neurons needs to be investigated further.

## Conclusion

In this report, we documented evidence that APP-BP1 plays a role in APP processing, especially in Aβ42 inhibition, in neuronal cells. First, we showed that APP-BP1 interacted with the APP processing enzyme, PS1, in brain tissues. Secondly, APP-BP1 suppression by siRNAs triggered a dramatic increase in Aβ42 levels in primary neurons. Thirdly, increased APP-BP1 expression specifically inhibited APP processing into Aβ in neurons. Lastly, we examined the potential mechanisms of APP-BP1 function in Aβ42 downregulation and provided evidence that APP-BP1 might downregulate Aβ42 levels by facilitating PS1-CTF degradation. Our data also suggest that a modified form of APP-BP1 interacts with PS1 in a membrane compartment where it plays a role in Aβ42 inhibition. Future research may determine how this pathway can provide therapeutic targets for Alzheimer's disease.

## Methods

### Antibodies

The following antibodies were used: rabbit anti-PS1 (AB5308) and 22C11 (Chemicon), BP339 [2], mouse anti-APP-BP1 (BD Transduction Lab), 6E10 and 4G8 (Signet), rabbit anti-cyclin B1 (Santa Cruz), 9E10 (ATCC), rabbit anti-APLP1 and rabbit anti-APLP2 (both from Calbiochem), rabbit anti-nicastrin, rabbit anti-β-catenin and mouse anti-γ-tubulin (all three from Sigma). The following antibodies are generous gifts from others: rabbit 369 antibody against APP cytoplasmic tail from Dr. Sam Gandy, rabbit anti-PS1-C [19] from Dr. Hui Zheng, and rabbit anti-PS1-NTF from Dr. Huaxi Xu.

### Co-immunoprecipitation (Co-ip)

Co-ip of APP-BP1 with PS1 was performed using adult Sprague Dawley Rat brain lysates. Briefly, rat brain tissues were isolated and solublized in a buffer containing 20 mM Tris, pH 7.5, 1 mM MgCl_2_, 125 mM NaCl, 2% CHAPS, and the protease inhibitor cocktail (1:250) from Sigma. The rabbit anti-PS1 antibody (AB5308) was prebound to protein G agarose by rocking in the lysis buffer for two hours at 4°C. The controls were beads alone or beads prebound to the rabbit anti-cyclin B1. The antibody-bound beads were washed 1× with the lysis buffer and added to the soluble brain extracts. The mixture was rocked for 2 hours on ice. The immune complex was collected by centrifugation at 4°C, washed 4× with the lysis buffer, and resolved on a 7.5% SDS-PAGE gel. The protein was transferred to a nitrocellulose membrane, blocked with 6% milk/PBS, and probed with the rabbit anti-APP-BP1 antibody, BP339 [2] or rabbit anti-nicastrin.

Co-precipitation of PS1 with APP-BP1 was carried out essentially as described above except that the brain membrane fraction proteins were used. To prepare the membrane fraction, rat brains were first homogenized for 15 strokes in a Dunce homogenizer in Buffer 1 containing 10 mM Tris, pH7.4, 150 mM NaCl, 5 mM EDTA, pH 8, and 250 mM sucrose plus the protease and phosphatase inhibitor cocktail (1 mM PMSF, 1 mM NaVO4, 5 mM iodoacetamide, 10 mM β-glycerol phosphate, 50 mM sodium pyrophosphate, and 50 mM NaF). The homogenate was centrifuged for 10 min at 1500 × g at 4°C and the pellet was discarded. The supernatant was saved and centrifuged again for 30 min at 20800 × g at 4°C. After centrifugation, the pellet was washed once with the Wash Buffer (1 M KCl, 20 mM HEPES, pH 7.2, 2 mM EGTA, 2 mM EDTA, 2 mM DTT, and the inhibitor cocktail). Proteins in the pellet were extracted (membrane fraction) for 1 hr on ice with the Extraction Buffer (20 mM Tris, pH8, 1 mM MgCl2, 125 nN NaCl, 2% CHAPS, and the inhibitor cocktail). These membrane proteins were cleared by centrifugation at 20800 × g at 4°C for 1 hr before the primary antibody was added. The membrane fraction was incubated with BP339 pre-bound to protein G beads, beads alone, or BP339 pre-immune antibody. The immune complex was incubated at 37°C for 10 min before loading on to an SDS-PAGE gel. The blot was probed with rabbit anti-PS1 (AB5308), rabbit anti-PS1-NTF, or rabbit anti-nicastrin.

### Glycerol velocity gradient centrifugation

Membrane fractions from adult Sprague Dawley rat brains were isolated as described above. The post-nuclear, purified membranes were extracted with 2% CHAPS, 100 mM KCl, 20 mM Hepes at pH 7.2, 2 mM EGTA, 2 mM EDTA, 2 mM DTT containing protease inhibitor cocktail for 1 hr on ice. The soluble membrane proteins were applied to the top of a glycerol gradient. The glycerol gradient protocol was as described previously with minor modifications [53,54]. Briefly, 1 ml of membrane protein extracts was applied to the top of an 11 ml 10–40% linear glycerol gradient containing 25 mM Hepes pH 7.2, 150 mM NaCl and 0.5% CHAPS. Gradients were centrifuged for 15 hrs at 210,000 × g at 4°C. One ml fractions were collected from top to bottom. After adding SDS loading buffer to 24 μl of each fraction, the samples were heated for 10 min at 100°C before loading on to SDS-PAGE gels. The blots were incubated with antibodies against PS1 (AB5308), PS1-NTF, PS1-CTF (PS1-C) [19], APP-BP1 (BP339) [2], nicastrin, or β-catenin followed by ChemiGlow detection (Alpha Innotech).

### Primary neurons

Primary neurons were isolated from E18 rat embryonic brain cortices. The cortices were gently triturated with a fine-tipped transfer pipette right after dissection. Cells were immediately plated in the neurobasal medium supplemented with 2% B27 (both from Invitrogen), 0.5 mM glutamine, 1% FBS, 1% equine serum, and 1× of penicillin/streptomycin (all from Sigma) at a density of 3 or 4 × 10^6 ^per 60 mm dish or 4 × 10^5 ^per well in 24-well plate. The medium was replaced with the complete fresh neurobasal medium at 1.5 hours post plating. To maintain the culture, half of the medium was replaced with fresh medium every three days. Cells were used on day 6 or 7 after plating.

### siRNA expressions in primary neurons

siRNAs were expressed via the short hairpin RNA (shRNA) vector, pHSVGET, in primary neurons. To construct the pHSVGET vector, we inserted the tRNA^val ^promoter sequence [55,56] into the SalI-digested pHSVprPUC-eGFP (a gift from Dr. JF Neumaier) [57]. shRNAs are normally processed by the Dicer enzyme into siRNAs inside the cell. Each shRNA duplex was designed with the Invitrogen web site BLOCK-iT™ RNAi Designer program using the TCAAGAG as the loop sequence. The siRNA targeting sequences for BP1 were (a) 5'-GGTAGATATCCAGGAGTATCT-3' or (b) 5'-GCATTTCTTCGAGTGGTAAGA-3'. The APP siRNAs were directed at (a) 5'-GTGATGCCCTTCTCG TTCCTG-3' or (b) 5'-GCAGAAGATGTGGGTTCAAAC-3'. The control random siRNAs are misssense, (a) 5'-CTTCATAA GGGGCATAGCTA-3' or (b) Negative siRNA (Stratagene). All constructs were sequenced for verification before HSV-1 viral packaging. The (a) group of siRNA vectors was used in most of the experiments, but the results were verified with the (b) group of siRNA vectors.

### Packaging of replication-defective HSV-1 virus

HSV-1 is a neural tropic virus whose genome does not integrate with the host genome. All HSV-1 viruses were packaged using the 2-2 cell line as described by Lim et al [58]. The infectious unit (IU) of each siRNA virus in the pHSVGET vector was determined by infecting primary neurons for 15 hours with serial diluted stocks. After fixation in 4% paraformaldehyde for 20 min at room temperatures, eGFP positive cells were visualized by fluorescence microscopy. The mycAPP-BP1 open reading frame from pHSVprPUC [8] was transferred into the p1005+ vector. The p1005+ vector was the same as pHSVprPUC-eGFP except that the multiple cloning sites were modified. The mycAPP-BP1/p1005+ and the p1005+ vector were packaged into HSV-1 and titered by eGFP expression. The expression of mycAPP-BP1 was verified by western blots using the anti-myc antibody 9E10.

### Neuron infection and analyses of APP and APLPs by western blots

Primary neuronal cultures maintained in 60 mm dishes were infected at day 7 post plating at 1 IU per cell for protein expression, or at 0.5 IU per cell for siRNA knockdown analyses. At 14 to 15 hours after infection, cells were washed with cold 1× PBS and lysed in RIPA buffer (0.5% sodium deoxycholate, 0.1% SDS, 1% NP40, 5 mM EDTA, 150 mM NaCl, and 50 mM Tris-HCL, pH 8.0) containing the protease inhibitor cocktail (1:250), 1 mM PMSF, 5 mM iodoacetamide, 50 mM NaF, 1 mM Na_3_VO_4_, and 10 mM β-glycerol phosphate (all from Sigma). The lysates were briefly sonicated. After protein assays using the Pierce BCA kit, equal amount of proteins were boiled in the SDS sample loading buffer and analyzed with SDS-PAGE gels.

### Aβ ELISA and INF-γ ELISA

Aβ42 and Aβ40 ELISAs were performed using the conditioned medium as well as cell lysates prepared with RIPA buffer as described [59], according to the manufacturer's protocols (Biosource International). Cell-associated (from 50 μg of protein) and secreted (from 1/15 volume of conditioned medium) Aβ42 in primary neurons were determined by Aβ42 ELISA. Cell-associated (from 50 μg of protein) and secreted (from 1/30 volume of medium of conditioned medium) Aβ40 in primary neurons was determined by Aβ40 ELISA. The amount of Aβ in samples that expressed APP without any siRNAs was used as 100% of Aβ production to normalize all the other samples in respective experimental conditions. The Aβ ELISA assay was repeated with the Aβ ELISA kits from The Genetics Company. INF-γ ELISA was performed on 50 μg of protein for intracellular INF-γ or on 1/15 volume of conditioned medium for secreted INF-γ using protocols provided by the manufacturer (Biosource International). The pHSVGET vector-based siRNA transfer into primary neurons did not induce INF-γ response – no intracellular or secreted INF-γ was detected by IFN-γ ELISA compared to IFN-γ standards (Biosource International) (data not shown). Therefore, it is unlikely that the reduced protein expression observed by specific siRNAs was caused by stress-induced global shutdown of gene transcriptions.

### Immunoprecipitation and western blot analyses of Aβ

Primary neurons expressing human APP_695 _along with mycAPP-BP1 or vector control was lysed in Extraction Buffer (0.5% sodium deoxycholate, 0.1% SDS, 20 mM EDTA, 150 mM NaCl, 50 mM Tris, pH 8) containing protease and phosphatase inhibitor cocktail. Cell extract (150 ug) was subjected to immunoprecipitation with 6E10 using a protocol as described [60]. The immune complex pulled down by 6E10 was boiled in sample loading buffer for 10 min and resolved on a bicine-Tris gel (15% T/5% C) with 8 M urea according to Klafki et al. [61]. The blot was boiled in 1 × PBS for 3 minutes before incubation with the 4G8 antibody overnight. Standard Aβ40 and Aβ42 peptides were purchased from American Peptide.

### Quantitative western blot and statistical analyses

Quantitative western blot analyses were based on densitometry of usually three or more independent experiments using the Scion Image software (Scion Corporation). Band densities were measured on exposures of blots within a linear range. The specific protein levels per lane in equal area of space were determined by deducting the background per individual lane and by normalizing that density to the amount of γ-tubulin per respective lane. One tail or two tail (more stringent) *t*-Tests for two samples assuming unequal variance were performed on the normalized data using the EXCEL data analysis tool (Microsoft). The statistical significance was set at p < 0.05.

### Colocalization of APP-BP1 and PS1 by fluorescence microscopy

Primary neurons grown on glass coverslips were fixed in 4% paraformaldehyde for 40 minutes at room temperature. Cells were then permeablized in 0.1% Triton X-100 for 15 minutes and blocked in Blocking Buffer (1% BSA and 10% normal goat serum in 10 mM phosphate-buffered saline) for 15 minutes. Cells were then incubated with the primary antibodies against PS1 (AB5308, 1:250, v/v) and 9E10 or mouse anti-APP-BP1 (1 ug/100 ul) for 2 hours. Cells were then washed twice with the Blocking Buffer and incubated for 1 hr with the secondary antibodies: Alexa Fluor 488 goat anti-rabbit and Alexa Fluor 594 goat anti-mouse (Molecular Probes, 1:300, v/v). Images were collected under a 40× objective using a Nikon confocal microscope (D-Eclipse C1) or a Nikon epifluorescence microscope (Eclipse E600).

## Abbreviations

APP, amyloid precursor protein; AD, Alzheimer's disease; FL-full length; CTF, carboxyl terminal fragment; Aβ, amyloid beta; NTF, amino terminal fragment; PS1, presenilin-1; siRNA, small interfering RNA.

## Competing interests

A patent on using HSV-1-mediated siRNA transfer is filed, which may or may not incur any competing interests.

## Authors' contributions

YC conceptualized and designed all the experiments, generated the pHSVGET shRNA expression vector, and prepared this manuscript. AMB worked on the co-immunoprecipitation, ELISA, and glycerol gradient analyses. DLM and RLN made the p1005 HSV-1 protein expression vector and the APP mutant constructs. REM and WSTG characterized human brain tissues.

## Supplementary Material

Additional file 1 APP-BP1 interacted with APP at a region close to the plasma membrane and the Triton-insoluble and SDS-soluble fraction of APP-BP1 decreased with normal aging. Click here for file
