# Supplementary material for: APP-BP1 inhibits Aβ42 levels by interacting with Presenilin-1
Source: Mol Neurodegener. 2007 Feb 7;2:3. doi: 10.1186/1750-1326-2-3 (PMC1802080; doi:10.1186/1750-1326-2-3)
Supplement: Additional file 1 — APP-BP1 interacted with APP at a region close to the plasma membrane and the Triton-insoluble and SDS-soluble fraction of APP-BP1 decreased with normal aging. [file 1750-1326-2-3-S1.pdf]

Additional File 1

Figure 1. APP-BP1 binds to APP in a region close to the plasma membrane. We previously reported that APP-BP1 binds to the C-terminal 31 amino acids of APP (C31) (1,2). By mutagenesis and co-immunoprecipitation, we found that APP-BP1 also interacted with APP close to the membrane spanning region.

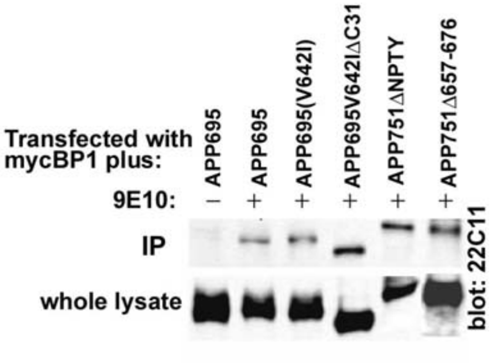

Method: COS cells were transfected with mycAPP-BP1 (mycBP1) along with an APP construct. All the expression plasmids used for transfections are in the pcDNA3 vector. The expression plasmids, mycAPP-BP1/pcDNA3 (1), APP_{695} and London mutation (V642I) (2), and Swedish mutation (Swe) (3) are all in the APP_{695} background and were transferred from the pHSVprPUC viral expression vector. APP_{695}V642IΔC31, APP_{751}ΔNPTY, and APP_{751}Δ(657-676) were constructed with proper primers using standard techniques. All constructs were verified by sequencing and by restriction endonuclease digests. Each sample was precipitated with the myc antibody 9E10 to bring down myc-APP-BP1 (mycBP1). Both the immunoprecipitation and the whole cell lysate blots were probed with 22C11. The negative control for co-precipitation was not transfected with mycBP1—no mycBP1 was present and therefore no APP was precipitated using the anti-myc antibody. In the sample where C31 of APP was deleted, mycBP1 was still able to precipitate APP (lane APP695V642IΔC31), indicating that APP-BP1 might interact with APP cytoplasmic tail close to the membrane insertion site.

Figure 2. APP-BP1 decreased with normal aging. APP-BP1 was isolated from the Triton-insoluble and SDS-soluble low density fractions extracted from the frontal cortex of postmortem human brain tissues from a younger group (50's) (n=4, mean age, 52.25 years old, PMI range=3-19h) and an older group (80's) (n=4, mean age=79.5 years old, PMI range=5-10h). Six μg of proteins were analyzed by Western blot using the APP-BP1 antibody BP339 and the mouse anti-γ-tubulin antibody. The average levels of relative protein density in each age group are reported, along with the standard error. APP-BP1 levels in the lipid-enriched fraction were lower in the older group, suggesting that there was a trend that APP-BP1 might decrease in normal aging (p=0.1, one-tail *t*-test). A statistical significance could not be reached at this time due to the limited availability of normal control cases.

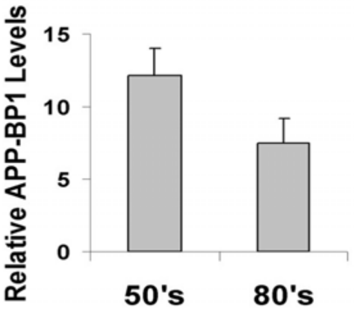

Methods: The Triton-insoluble and SDS-soluble lipid rafts was isolated from postmortem human brain tissues essentially as described before (2). Frozen postmortem human brain tissues were homogenized 20 for strokes in a Dounce homogenizer in Buffer T containing 1% Triton X-100, 0.32 M sucrose, 10 mM Tris, pH8.0, 1 mM PMSF, 1mM NaVa, 0.1 mM iodoacetamide, and Sigma inhibitor cocktail (1:250). The homogenized tissue was incubated on ice for 30 min before centrifugation for 6 min at 1085 x *g*. The Triton-insoluble pellet was washed once with Buffer T and then incubated for 30 min with Buffer S containing 1% SDS in 1xPBS plus the protease inhibitors as in Buffer T. After centrifugation at 20,800 x *g*, the Triton-insoluble and SDS-

soluble protein extracts were transferred to a new tube (these were the crude lipid rafts). Protein concentrations were determined with the Pierce BCA protein assay kit. Equal amount of proteins were analyzed by Western blotting.

References

1. Chow, N., Korenberg, J. R., Chen, X. N., and Neve, R. L. (1996) *J Biol Chem* **271**(19), 11339-11346
2. Chen, Y., Liu, W., McPhie, D. L., Hassinger, L., and Neve, R. L. (2003) *J Cell Biol* **163**(1), 27-33
3. McPhie, D. L., Lee, R. K., Eckman, C. B., Olstein, D. H., Durham, S. P., Yager, D., Younkin, S. G., Wurtman, R. J., and Neve, R. L. (1997) *J Biol Chem* **272**(40), 24743-24746
